# Traumatic Porto-Mesenteric Venous Gas: Emergency Computed Tomography Findings

**DOI:** 10.1016/j.acepjo.2026.100335

**Published:** 2026-02-10

**Authors:** Wang Xiaqing, Fang Hui

**Affiliations:** Department of Radiology, Shanghai Pudong Hospital, Shanghai, China

**Keywords:** porto-mesenteric venous gas (PMVG), blunt abdominal trauma, emergency computed tomography, pneumatosis intestinalis, emergency radiology

## 1 Patient Presentation

A 30-year-old woman presented to the emergency department 90 minutes after a motor vehicle crash (blunt head/chest/abdominal trauma). Emergency computed tomography (CT) was performed to evaluate traumatic injuries, revealing 4 key findings with critical emergency implications as follows:

1. Axial head CT: diffuse cerebral edema (hypodense parenchyma and effaced sulci) indicated traumatic brain injury—guiding immediate intracranial pressure management (eg, mannitol administration) (Fig 1).

**Figure 1:**
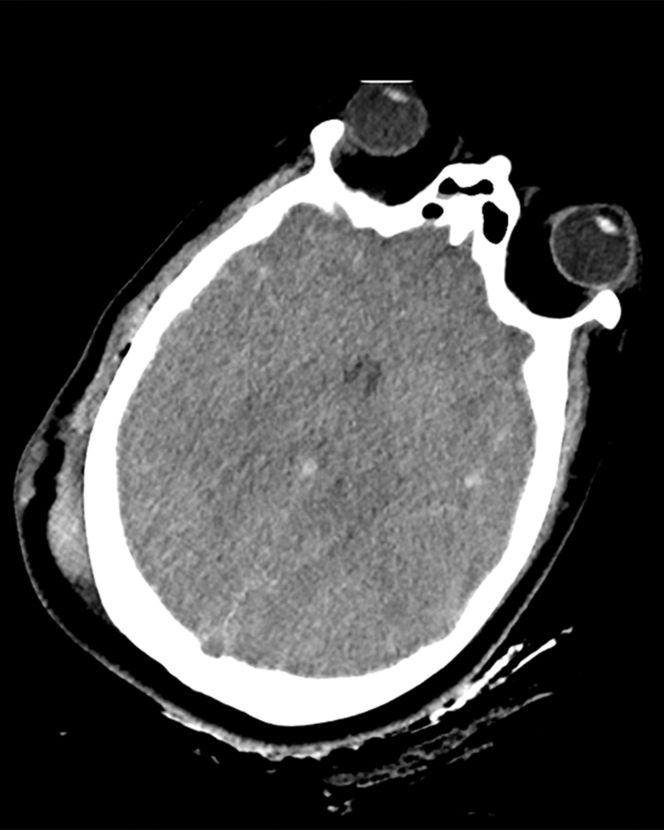
Axial head computed tomography: diffuse cerebral edema (hypodense parenchyma, effaced sulci) indicated traumatic brain injury.
2. Axial abdominal CT: focal gas foci in the right hepatic vein confirmed hepatic venous pneumatosis, a core component of porto-mesenteric venous gas (PMVG). Isolated hepatic venous gas may be transient, but its coexistence with other abdominal findings elevated clinical suspicion (Fig 2).

**Figure 2:**
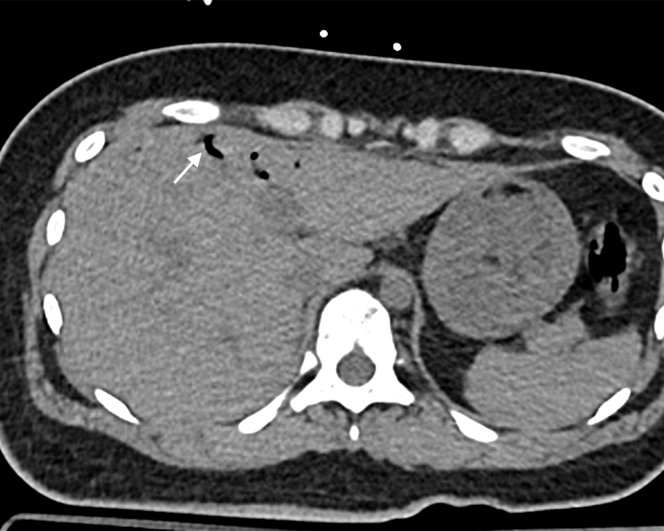
Axial abdominal computed tomography: hepatic venous pneumatosis (arrow, left hepatic vein). Focal gas foci in the right hepatic vein confirmed hepatic venous pneumatosis, a core component of porto-mesenteric venous gas.
3. Coronal abdominal CT: linear gas tracks in superior mesenteric vein branches extended PMVG beyond the hepatic veins, ruling out isolated venous gas and suggesting more extensive gastrointestinal involvement (Fig 3).

**Figure 3:**
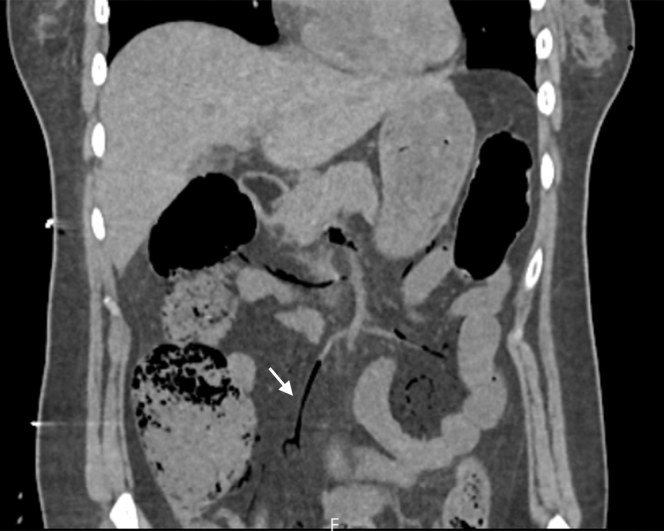
Coronal abdominal computed tomography: mesenteric venous pneumatosis (arrow, superior mesenteric vein branches). Linear gas tracks in superior mesenteric vein branches extended porto-mesenteric venous gas beyond the hepatic veins, ruling out isolated venous gas and suggesting more extensive gastrointestinal involvement.
4. Axial abdominal CT: submucosal gas in the ascending colon (colonic pneumatosis intestinalis) was the critical high-risk feature—this finding, combined with PMVG and hypotension, strongly predicted bowel ischemia, prompting urgent surgical consultation (Fig 4).

**Figure 4:**
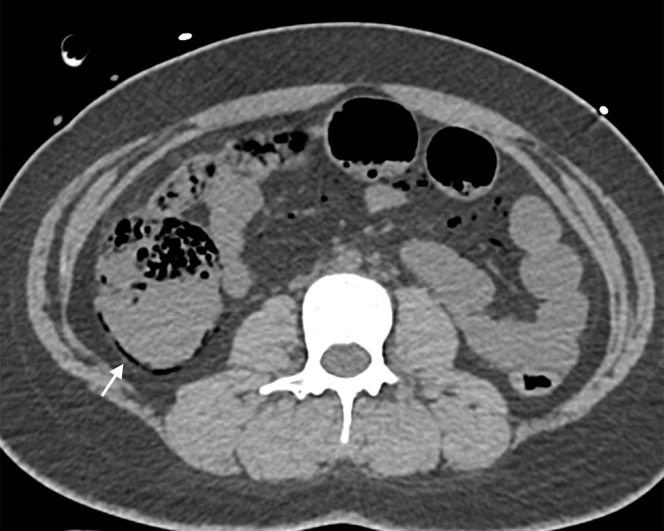
Axial abdominal computed tomography: colonic pneumatosis intestinalis (arrow, ascending colon) was the critical high-risk feature—this finding, combined with porto-mesenteric venous gas and hypotension, strongly predicted bowel ischemia, prompting urgent surgical consultation.

## 2 Key Results

In trauma patients, PMVG on emergency CT may indicate either transient gas entrapment or subtle, life-threatening bowel injury—emphasizing the need for correlation with clinical status (eg, hemodynamics and lactate) to avoid mismanagement.

Emergency CT findings, specifically the coexistence of PMVG with colonic pneumatosis intestinalis, stratify traumatic PMVG as high-risk for bowel ischemia, warranting urgent surgical consultation.

## 3 Introduction

A 30-year-old woman with no medical history was brought to the emergency department (ED) 90 minutes after a motor vehicle crash (blunt trauma to head, chest, and abdomen). On arrival to the ED, the woman’s blood pressure was 90/55 mm Hg, heart rate was 122 beats/min, respiratory rate was 26 breaths/min, and Spo_2_ was 91% (room air). She was somnolent (Glasgow Coma Scale 9: E2V2M5), with no verbal report of abdominal pain, but physical examination showed mild abdominal distension and diffuse tenderness.

## 4 Emergency Clinical Decision Making

Given PMVG + pneumatosis intestinalis + hypotension (systolic blood pressure <90 mm Hg), the ED team suspected acute bowel ischemia and recommended urgent exploratory laparotomy. The patient’s family declined surgery, citing concern for neurologic and respiratory instability.

## 5 Outcome

Within 2 hours of PMVG detection, the patient developed refractory hypotension (requiring norepinephrine of 0.8 μg/kg/min) and multiorgan failure. She died shortly after; no autopsy was performed.

## 6 Discussion

Traumatic PMVG arises from 2 emergency-relevant mechanisms: (1) trauma–induced gastrointestinal mucosal injury (disrupting the intestinal barrier) and (2) posttraumatic intestinal distension with elevated intraluminal pressure (driving gas into the portal venous system).^1^^,^^2^ A multicenter study of 290 patients confirmed that PMVG + pneumatosis intestinalis elevates 90-day mortality to 67.2%—a rate far higher than pneumatosis intestinalis alone (46.5%)—consistent with this case’s fatal outcome without surgery.^3^

In the ED, emergency CT is critical for stratifying traumatic PMVG: isolated PMVG may occasionally correspond to benign, reversible conditions,^3^ but PMVG with pneumatosis intestinalis requires immediate surgical consultation.^1^^,^^2^ This aligns with clinical evidence that traumatic PMVG rarely resolves spontaneously when combined with intestinal gas signs—delayed intervention increases the risk of irreversible bowel necrosis and multiorgan failure, as seen in this case.^1^ Additionally, emergency CT’s ability to distinguish isolated hepatic venous gas from extensive PMVG (involving mesenteric veins) helps avoid overtreatment of benign cases while ensuring high-risk patients receive timely surgical evaluation.^2^

## 7 CT Technique

GE Revolution CT; 120 kVp, 200 mAs, 1-mm slices; reconstructed in axial and coronal planes. Image annotation: Solid color arrows (1.0 pt) for clear identification of pathologic findings.

## Funding

This research was funded by Project Approval of Special Disease Construction Project of Shanghai Pudong Hospital (grant Tszb2023-08) and Discipline Construction Project of Shanghai Pudong New Area Health Commission (grant PWZbr2022-16).

## Conflict of Interest

All authors report no conflicts of interest relevant to this work.
